# Disrupted self-referential processing and empathy-based interventions in mental disorders: neural mechanisms, cognitive biases, and therapeutic integration

**DOI:** 10.3389/fpsyt.2026.1850823

**Published:** 2026-07-06

**Authors:** Siqintu, Qinglin Bao, Dezhi Yang, Zhiheng Dong, Chula, Husile, Muxiyanle, Zhiyong Bao, Limei He, Wulan, Temuqile

**Affiliations:** 1 College of Mongolian Medicine and Pharmacy, Inner Mongolia Medical University, Hohhot, China; 2 International Mongolian Hospital of Inner Mongolia, Hohhot, China; 3 Department of Pharmacy, Affiliated Hospital of Inner Mongolia Medical University, Hohhot, China

**Keywords:** anxiety disorders, depression, empathy-based intervention, neural mechanisms, schizophrenia, self-referential processing, social cognition

## Abstract

Self-referential processing (SRP), the mental activity of linking experiences and stimuli to the self, is fundamental to shaping identity, regulating affect, and navigating complex social environments. Disruptions in SRP are increasingly recognized as a transdiagnostic hallmark across psychiatric disorders, including schizophrenia spectrum disorders, major depressive disorder, and anxiety disorders. Such disruptions often manifest as distorted self-perception, negative cognitive-affective biases, and dysfunctional connectivity within key neural networks, particularly the default mode network (DMN) and the medial prefrontal cortex (mPFC). Emerging evidence reveals a dynamic bidirectional relationship between SRP and empathy. Impairments in self-processing may hinder the capacity to accurately infer others’ emotional and cognitive states, while deficits in empathic attunement may reinforce maladaptive self-focus. This interplay contributes to social withdrawal, interpersonal dysfunction, and emotional dysregulation across diagnostic boundaries. Recent therapeutic advances suggest that interventions targeting the interface between SRP and empathy may effectively modulate shared neurocognitive mechanisms. Such interventions include metacognitive therapy, mindfulness-based interventions, and psychedelic-assisted psychotherapy. These therapeutic approaches show preliminary but promising evidence for reducing maladaptive self-focused attention, enhancing emotion regulation, and improving social functioning. This review synthesizes empirical evidence regarding the neural underpinnings of SRP, delineates disorder-specific alterations in self-referential processing, and examines the bidirectional interplay between SRP and empathic functioning. We further outline a conceptual framework for empathy-based interventions targeting SRP dysfunction and propose future directions for precision psychiatry research. Emphasis is placed on developing personalized, mechanism-driven therapeutic strategies informed by computational modeling, transdiagnostic biomarker identification, and digital therapeutic platforms.

## 1 Introduction

Social cognitive deficits are increasingly recognized as fundamental transdiagnostic features across a broad spectrum of psychiatric disorders, including major depressive disorder and anxiety disorders (1, 2). Among these critical social cognitive domains, self-referential processing (SRP) assumes a pivotal role in constructing and maintaining coherent self-identity, interpreting affective experiences, and regulating complex social behaviors. SRP encompasses the comprehensive set of cognitive operations involving the relationship between external or internal stimuli and oneself, thereby supporting essential psychological processes including self-evaluation, autobiographical memory retrieval, and introspective reflection (3, 4).

Neuroimaging investigations have consistently demonstrated that SRP is mediated by a distributed network of brain regions, with particular emphasis on the medial prefrontal cortex (mPFC), ventromedial prefrontal cortex (vmPFC), and constituent regions of the default mode network (DMN), including the posterior cingulate cortex (PCC) and precuneus (5, 6). Aberrant neural activity or altered connectivity patterns within these critical regions have been systematically associated with diverse psychopathological symptoms, including negative self-schematic processing, ruminative thought patterns, and impaired self-other differentiation (7, 8). In schizophrenia spectrum disorders, SRP dysfunction may manifest as impaired self-boundary processing and agency misattribution (9), whereas depressive conditions are frequently characterized by excessive self-focused attention and negative valence bias (10, 11).

Empathy, conceptualized as the capacity to understand and share the emotional states of others, shares substantial overlapping neurocognitive substrates with SRP, such that dysfunction in either system may exacerbate deficits in the complementary domain (12, 13). Therapeutic interventions that simultaneously modulate these interconnected systems—including metacognitive reflection and insight therapy (MERIT) approaches, mindfulness-based therapies, and psychedelic-assisted psychotherapy—have shown preliminary but encouraging evidence of improvements in improving emotional regulation (ER) and social functioning outcomes (14–17).

**Figure 1:**
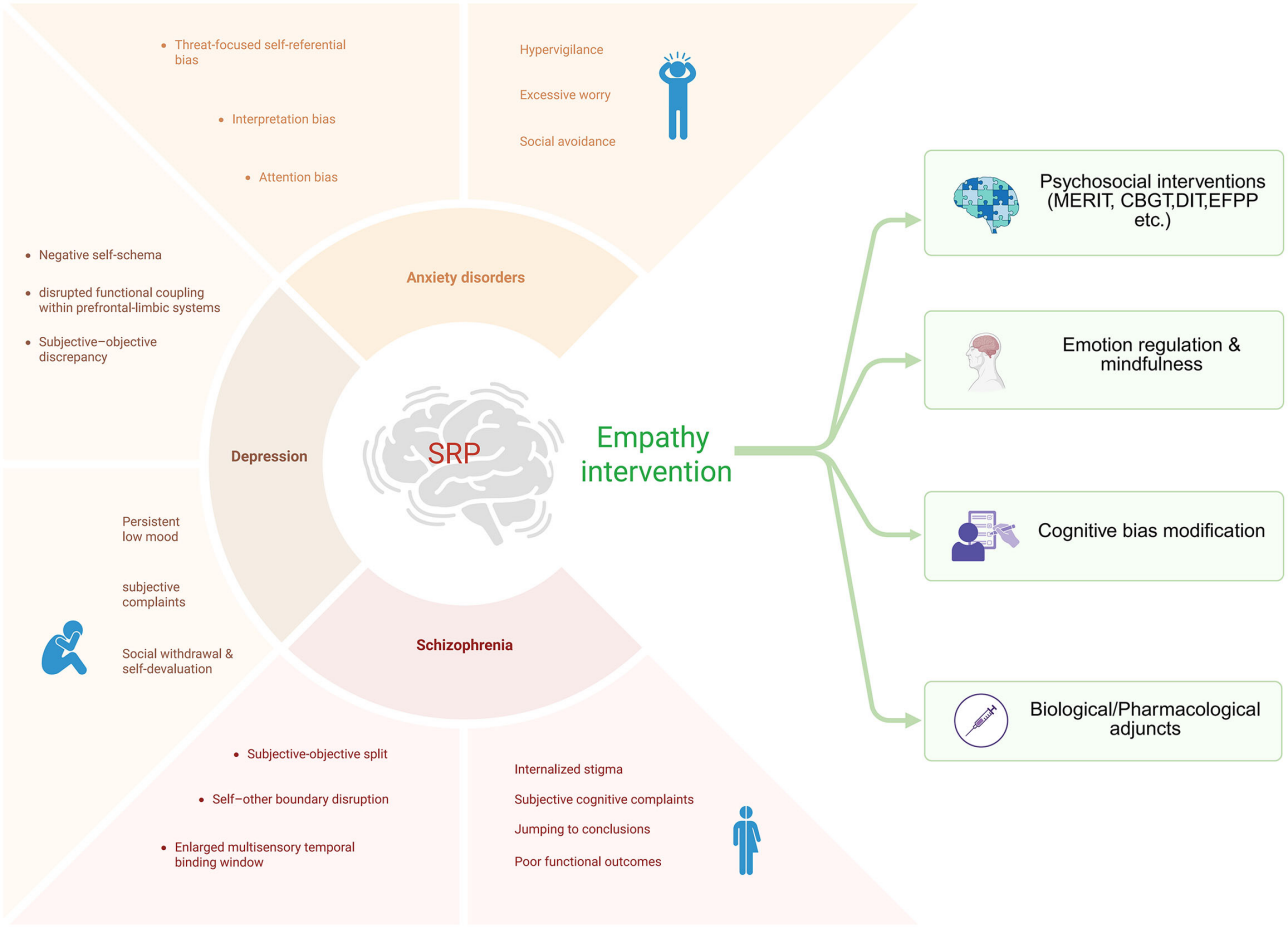
Integrative conceptual framework linking self-referential processing, empathy, disorder-specific manifestations, and intervention targets in psychiatric disorders. This figure synthesizes the relationships discussed across Sections 2–5 and is intended as an organizational schematic rather than a model in which all depicted pathways have been empirically validated.

In this comprehensive review, we synthesize recent empirical progress concerning the neural and cognitive underpinnings of SRP, characterize its disorder-specific manifestations across major psychiatric conditions, and examine the bidirectional interaction between SRP and empathic functioning. Furthermore, we outline the empirical foundation for emerging empathy-based interventions and propose future directions for precision psychiatry and transdiagnostic treatment strategies targeting the critical SRP-empathy interface (**Figure 1**). It should be noted that the current evidence base for many of these interventions remains at an early stage, and the degree of empirical support varies considerably across therapeutic modalities and diagnostic populations.

### 1.1 Conceptual differentiation: self-referential processing and related constructs

The present review is organized around the construct of SRP; however, several related but distinct constructs—metacognition, meta-awareness, self-reflection, and self-focused attention—frequently appear in the broader literature and risk conflation with SRP if not explicitly differentiated. The following definitions are intended to establish terminological precision for the remainder of this review.

SRP refers to the cognitive process by which external or internal stimuli are evaluated in relation to the self. It is operationally indexed by preferential neural activation in the DMN—particularly the mPFC and PCC—during self-referential tasks, and at the behavioral level by the self-referential memory advantage: the well-established finding that information encoded with reference to the self is subsequently recalled more accurately than information encoded with reference to others (18, 19). Critically, SRP can operate in an automatic, implicit fashion without requiring deliberate introspection.

Metacognition, by contrast, refers to higher-order knowledge about and monitoring of one’s own cognitive processes—for example, judging one’s memory accuracy or regulating one’s problem-solving strategies. Metacognition is more evaluative and explicitly controlled than SRP, and while the two constructs interact (e.g., self-referential encoding can draw on metacognitive monitoring), they are conceptually distinguishable and may rely on partially dissociable functional and neural processes.

Meta-awareness denotes the explicit recognition that one is currently in a particular mental state—for instance, becoming aware that one is ruminating. It is a momentary, phenomenological event that presupposes but is not equivalent to SRP.

Self-reflection refers to deliberate, sustained introspective examination of one’s own thoughts, feelings, motivations, and experiences. Unlike SRP, self-reflection is intentional, effortful, and typically verbally mediated. It may be facilitated by intact SRP but is not reducible to it.

## 2 Neural basis of SRP

### 2.1 Functional architecture of SRP

SRP is supported by a distributed neural network anchored in the DMN—a large-scale system encompassing the mPFC, PCC, and precuneus, among other regions (20, 21). Within this network, the mPFC serves as a core hub, and its ventral subregion—vmPFC—plays a particularly prominent role in SRP. Functional neuroimaging studies have repeatedly shown that DMN midline regions, particularly the mPFC/vmPFC and PCC, are engaged during self-referential tasks, such as evaluating personality traits and reflecting on personal experiences (6, 22).

The vmPFC is particularly implicated in the self-prioritization effect, where self-related stimuli are processed more efficiently than those related to others. For instance, Yin et al. (5) demonstrated that applying inhibitory transcranial direct current stimulation to the vmPFC effectively abolished the behavioral self-prioritization effect (5). This finding confirms a causal role for this region in modulating self-related attention and working memory processes.

Moreover, the neural representation of self-esteem appears to be closely associated with vmPFC activity patterns. Individuals with higher trait self-esteem show stronger attentional biases toward self-related stimuli and increased vmPFC activation. This suggests that the vmPFC encodes stable, trait-like self-evaluations (23). Collectively, these findings suggest the vmPFC facilitates real-time detection of self-relevance and maintains long-term evaluative structures central to self-concept formation. These neural properties carry direct clinical implications. In major depressive disorder, vmPFC and mPFC hyperactivation during SRP tasks has been consistently associated with ruminative self-focused cognition and the consolidation of negative self-schemas (10, 24). Clinically, this pattern maps onto the hallmark symptoms of worthlessness and self-criticism: the same midline circuitry that normally supports adaptive self-evaluation becomes chronically biased toward negative self-relevant content, rendering neutral or ambiguous stimuli more likely to be encoded as self-threatening. Conversely, in schizophrenia, aberrant vmPFC connectivity may undermine the stability of self-representations, contributing to the self-boundary disturbances and agency misattribution described in Section 3.1. Understanding mPFC and vmPFC dysfunction in these terms—not merely as imaging findings but as neural substrates of specific psychopathological experiences—is essential for designing mechanism-targeted interventions.

### 2.2 Electroencephalography and oscillatory dynamics

Electrophysiological studies have helped characterize the temporal dynamics of self-referential processing (SRP). In a trait-judgment paradigm comparing self- with other-referential evaluation, Mu and Han (25) showed that self-referential judgments were associated with induced (i.e., non-phase-locked) increases in theta and alpha power, alongside decreases in beta and gamma power, with partially dissociable temporal and topographic distributions (25).

Detailed analyses by Mu and Han (25), based on non-phase-locked oscillatory activity during positive versus negative trait judgments, have shown that the emotional valence of traits predominantly influences neural activity in the theta and alpha frequency bands, whereas self-relatedness exerts stronger effects on activity in the beta and gamma frequency bands. These findings suggest that non–phase-locked neural activity plays a crucial role in self-referential processing, with low-frequency and high-frequency oscillations potentially corresponding to distinct emotional and cognitive components of processing self-relevant information. These frequency-band distinctions may carry functional implications relevant to psychopathology, although these interpretations remain provisional and should not be treated as direct clinical markers. Theta synchronization (4–8 Hz) is understood to reflect the integration of self-relevant information with existing autobiographical memory networks—a process that, when dysregulated, may underlie the over-consolidation of negative self-memories characteristic of depression. Alpha suppression (8–12 Hz) indexes the selective engagement of attentional resources with self-relevant content; pathologically elevated alpha suppression in response to negative self-referential stimuli may reflect the attentional capture by self-critical material seen in ruminative disorders. Beta and gamma activity (13–80 Hz), associated here with self-relatedness rather than valence, may subserve higher-order self-monitoring and metacognitive operations—processes implicated in the impaired self-other differentiation observed in schizophrenia spectrum disorders.

Mu and Han (25) specifically demonstrated that non-phase-locked (induced) oscillatory activity—including event-related synchronization in theta and alpha and desynchronization in beta and gamma bands—differentiates self- from other-referential processing. Although phase-locked (evoked) responses also accompany self-referential judgments as evidenced by ERP research, Mu and Han (25) did not analyze phase-locked components; therefore, the claim that SRP relies on both types of neural activity should be understood as drawing on the broader oscillatory and ERP literatures in combination. Importantly, these oscillatory patterns are not exclusive to SRP and are observed across numerous cognitive domains. From a clinical standpoint, the dominance of non-phase-locked, endogenously driven oscillatory activity in SRP is particularly significant: it suggests that self-referential thought is not merely time-locked to external stimuli but involves sustained, internally driven cognitive processing. It should be noted that this conclusion is drawn from task-evoked oscillatory data; the relationship between these endogenous dynamics and resting-state neural activity requires independent evidence, such as the MEG-DMN correspondence reported by Brookes et al. (26), who showed that MEG-derived oscillatory power envelopes in the alpha and beta bands closely track the spatial and temporal dynamics of the DMN (26). This endogenous quality provides a neural basis for understanding why rumination and intrusive self-focused ideation—both of which arise in the absence of external triggers—are so persistent and treatment-resistant in depression and anxiety.

### 2.3 Computational and network models

Beyond regional activation patterns, network-based and computational models have yielded deeper insights into the organizational principles that govern SRP. The DMN can be functionally subdivided into two core subsystems: a cortical midline subsystem (CMS), which includes the mPFC and PCC and is primarily involved in self-evaluative and reflective thought, and a parietotemporal subsystem (PTS), which is implicated in autobiographical memory retrieval and spatial self-representation (6).

Meta-analytic investigations have shown that CMS regions are more selectively involved in SRP than PTS regions, suggesting functional specialization within the broader DMN architecture. Recent advances have also integrated structural and functional neuroimaging data to propose a multistable self–other processing network. Using sophisticated fMRI meta-analytic techniques combined with diffusion spectrum imaging methodologies, Chen and Huang (27) identified a candidate neural network that simulates gamma-band multistability, thereby supporting the flexible switching between self-oriented and other-oriented mental states.

This computational approach provides one illustrative mechanistic model for how self–other boundaries may be dynamically maintained, but directly comparable computational studies of SRP remain limited. Taken together, these findings provide a provisional multilevel framework for SRP, although the computational evidence base remains comparatively sparse.

The neuroimaging and electrophysiological findings reviewed in this section converge to support a distributed neural model of SRP; however, several methodological limitations warrant caution in interpretation. First, the majority of studies employ cross-sectional designs, precluding causal inference about whether observed neural differences precede or follow psychopathological symptoms. Second, considerable heterogeneity exists in task paradigms across studies—verbal trait-judgment tasks, pictorial stimuli, and autobiographical recall tasks likely engage overlapping but non-identical neural circuits, limiting strict cross-study comparisons. Third, most neuroimaging samples are relatively small (typically N < 50), raising concerns about statistical power and replicability. Fourth, the functional specificity of DMN activation to SRP has been questioned, given that the DMN is recruited across numerous cognitive domains including episodic memory retrieval and prospective thinking. Findings that implicate DMN hyperactivity as a marker of SRP dysfunction should therefore be interpreted as preliminary until replicated with larger, pre-registered samples using standardized paradigms.

## 3 Disorder-specific aberrations in SRP

### 3.1 Impaired self-perception in schizophrenia

In schizophrenia spectrum disorders, impairments in SRP are characterized by a profound dissociation between subjective self-perception and objective cognitive performance. Empirical studies consistently show that patients often overreport cognitive difficulties. These subjective reports have weak or no correlation with performance on standardized neuropsychological assessments (28, 29). Notably, elevated subjective complaints correlate more strongly with affective symptoms (e.g., anxiety and depression) than with actual cognitive deficits.

This perceptual discrepancy is further compounded by prominent cognitive biases. One notable example is the “jumping to conclusions” bias, in which patients make hasty decisions based on minimal evidence, especially when processing self-relevant or ambiguous stimuli (30). Additionally, SRP dysfunction interacts with aberrant salience attribution mechanisms, contributing to the development and maintenance of delusional ideation.

At the level of self-boundary integration, patients show a markedly impaired ability to differentiate between representations of self and others. Sandsten et al. (9) used the Enfacement Illusion paradigm to demonstrate that patients with schizophrenia have increased susceptibility to incorporating external stimuli into their self-representation, even under asynchronous visuotactile stimulation conditions. This finding suggests a widened multisensory temporal binding window, which facilitates abnormal self–other blending phenomena. Such distortions in embodied self-processing may underlie core psychotic symptoms, including thought insertion and delusional misidentification syndromes.

### 3.2 Characteristics of self-referential processing in depression

In major depressive disorder, SRP alterations are characterized by pervasive negative self-schematic processing and an increased dissociation between subjective and objective performance. Self-referential encoding task (SRET) provides direct evidence of SRP disruption in depression. Dainer-Best et al. (19) demonstrated in a large-scale sample that individuals with depression endorse significantly more negative and fewer positive self-descriptors compared with controls, and show a reduced memory advantage for positively valenced self-referenced words—reflecting a systematic negative bias in self-referential encoding (19). Similarly, Lou et al. (31) reviewed converging evidence that abnormal self-knowledge in major depressive disorder is characterized by preferential processing of negative self-relevant information and attenuated processing of positive self-relevant material (31).

Functionally, researchers have observed abnormal activation patterns in the rostral anterior cingulate cortex (rACC) and the amygdala during self-referential processing tasks. Wagner et al. (10) reported that individuals with depression did not show valence-specific modulation in the rACC. They also found that even neutral self-relevant stimuli activated the amygdala and reward-related regions. Post-task assessments revealed reduced activity in the cognitive control network, suggesting that SRP dysfunction impairs executive regulatory mechanisms.

Studies of emotional word processing further support this pathological profile. Renner et al. (11) and Preglej et al. (32) found that patients with depression were more likely to endorse negative emotional traits and reject positive ones as self-descriptive. This pattern was linked to hypoactivation in medial and dorsolateral prefrontal cortical regions. These convergent findings support a model of SRP in depression characterized by affective interference, prefrontal–limbic dysregulation, and persistent negative self-representation.

### 3.3 Self-related cognitive biases in anxiety disorders

Patients with anxiety disorders also exhibit significant SRP-related distortions, particularly in the form of fear-congruent cognitive biases. In social anxiety disorder (SAD), Button et al. (33) demonstrated that highly anxious individuals learned negative self-related rules (e.g., “I am disliked”) more readily than positive ones. This suggests a systematic bias toward internalizing negative social evaluations.

Attentional biases further compound these pathological effects. Event-related potential (ERP) studies show that individuals with high social anxiety exhibit increased early attentional processing (elevated P1 amplitude) and reduced structural face processing (lower N170 amplitude) when viewing others’ emotional expressions, especially angry faces (34). These attentional distortions are specific to socially threatening stimuli. They may reinforce maladaptive self-appraisal patterns.

Research in health anxiety (i.e., a clinical condition characterized by excessive preoccupation with having or developing a serious illness, distinct from healthy individuals with elevated anxiety traits) shows that interpretation bias, particularly the tendency to catastrophize benign somatic symptoms, is the strongest predictor of symptom severity (35). In this context, interpretation bias may engage self-referential evaluative processes, because ambiguous bodily sensations are appraised as personally threatening and therefore acquire exaggerated self-relevance. These findings underscore the multifaceted nature of SRP dysfunction in anxiety disorders, encompassing attentional, interpretive, and memory-related cognitive distortions.

Collectively, these disorder-specific profiles share a common thread: disruption of self-referential processing mechanisms, manifesting with distinct symptom-level expressions across diagnoses.

The disorder-specific SRP profiles described above rest on a literature with several recurring limitations. First, reliance on self-report measures—particularly for subjective cognitive complaints and self-appraisal biases—introduces significant method variance and conflates SRP with metacognitive evaluation, as noted in Section 1.1. Studies using direct behavioral indices of SRP (e.g., SRET paradigms) remain comparatively sparse, particularly in anxiety disorders. Second, heterogeneity in depression subtypes (melancholic, atypical, treatment-resistant) is rarely accounted for in SRP research, yet preliminary evidence suggests that DMN connectivity alterations differ across subtypes—a finding that complicates transdiagnostic generalization. Third, most studies reviewed here involve clinical samples from single sites with limited demographic diversity, constraining the external validity of findings. Fourth, the direction of effects is rarely tested: it remains unclear whether SRP dysfunction precedes the onset of psychiatric symptoms, emerges concurrently, or represents a consequence of chronic illness. Longitudinal and experimental designs are needed to resolve these directional questions before SRP dysfunction can be confidently positioned as a causal mechanism rather than a correlate.

## 4 Empathy and self-processing: bidirectional links

Before reviewing the evidence for bidirectional SRP-empathy interactions, a methodological note is warranted. As delineated in Section 1.1, SRP is conceptually distinct from related constructs such as metacognition, self-reflection, and mentalizing, although these processes interact in practice and share partially overlapping neural substrates. In the sections that follow, several lines of evidence derive from studies that operationalize their key variables in terms of self-reflectiveness (a component of metacognition) or mentalizing (a broader construct encompassing both self- and other-directed mental state attribution) rather than SRP per se. Where such evidence is cited, it is used to illuminate processes that are theoretically adjacent to, and likely engage, self-referential mechanisms—but readers should bear in mind that the inferential bridge from these constructs to SRP proper involves assumptions that remain to be empirically verified.

### 4.1 Neurocognitive and behavioral interaction mechanisms

Empathy and self-referential processing interact to form a multi-layered psychobiological mechanism that integrates both affective and cognitive dimensions. Self-referential processing is not merely a detached cognitive function. Instead, it is strongly linked to affective evaluation processes. Empirical research suggests that spontaneous self-referential coding can significantly influence the cortical processing of emotional stimuli, even without explicit self-referential instructions (36). Based on Herbert et al. (36), it has been hypothesized—rather than established—that this influence operates via phasic autonomic arousal mechanisms that enhance the encoding of self-relevant stimuli. This enhanced encoding, in turn, contributes to the formation of subjective emotional experiences and the consolidation of autobiographical memory traces.

Emerging evidence indicates a fundamental link between self-referential cognition and empathic capacity. This relationship is particularly pronounced in individuals with autism spectrum conditions (ASC). Research indicates that individuals with ASC exhibit simultaneous deficits in self-referential processing and empathic accuracy (37). This finding aligns with simulation theories suggesting that self-referential mechanisms are foundational to empathic functioning. Consequently, empathy should not be viewed merely as the passive interpretation of others’ emotional states, but rather as an emergent property arising from the dynamic interplay between self-referential networks and distributed socio-cognitive circuits. It should be noted that the ASC example is employed here as a theoretical proof-of-concept for simulation theory, and that co-occurring deficits in ASC do not necessarily imply the same mechanistic linkage in schizophrenia, depression, or anxiety. Generalization across clinical populations requires empirical verification.

Meta-analytic neuroimaging studies have consistently implicated cortical midline structures, including medial prefrontal regions, in self-referential processing (18). In parallel, meta-analytic work on empathy has identified the anterior insula and cingulate cortex as core components of affect-sharing networks (38). However, empathy depends not only on affective resonance but also on maintaining an appropriate distinction between self and other, as emphasized in influential neurocognitive models of empathy (39). Consistent with this view, experimental work has shown that emotional egocentricity bias is reduced by neural mechanisms supporting self–other distinction, particularly in the right supramarginal region (40). Taken together, these findings suggest that effective empathic responding depends on dynamic coordination between self-referential systems and neural mechanisms that regulate self–other differentiation.

### 4.2 Influence of self-reflectiveness on empathy

Empathic abilities are closely linked to self-referential processes across both clinical and non-clinical populations. In schizophrenia spectrum disorders, the relationship between empathy deficits and distress tolerance appears to be moderated by—rather than uniformly exacerbated by—impairments in metacognitive self-reflectiveness. As noted in Section 1.1, self-reflectiveness is a metacognitive construct that is related to but not synonymous with SRP; we draw on this finding here because self-reflective monitoring likely engages and depends upon intact self-referential mechanisms, although this link has not been directly tested. Bonfils et al. (16) in a study of 54 individuals with schizophrenia, found that bivariate correlations among empathy, distress tolerance, and self-reflectiveness were non-significant; however, moderation analyses revealed a significant interaction effect (16). Specifically, among patients with lower self-reflectiveness, reduced distress tolerance was associated with lower empathy, whereas no such association was observed in individuals with higher self-reflectiveness. These findings suggest that greater self-reflectiveness may buffer against the detrimental effects of low distress tolerance on empathic functioning.

Similar patterns have been observed in the general population. A large-scale study (N = 2376) demonstrated that self-reported empathy showed only weak correlations with behaviorally assessed social cognition. This dissociation points to the importance of self-directed emotional processing at the SRP-empathy interface: failures in identifying and labeling one’s own affective states may reflect one aspect of disrupted self-referential emotional processing, making alexithymia a construct of direct theoretical relevance here. Accordingly, Sunahara et al. (17) found that alexithymia—defined as difficulty identifying and describing one’s own emotions—was more strongly associated with both social-cognitive performance and affective sharing than self-reported empathy measures such as perspective-taking and emotional contagion (17). These findings underscore the central role of self-awareness and emotional clarity in empathic and social-cognitive processes.

Although the clinical populations discussed in this review are the primary focus, non-clinical evidence can help clarify the basic mechanisms linking SRP to empathic behavior. As one illustrative example, a study of medical students found that higher scores on the Jefferson Scale of Empathy–Student Version were associated with slightly higher ratings of facial expressivity during clinical interactions with standardized patients, whereas verbal empathy ratings did not differ (41). These results indicate that self-referential appraisals of empathic capacity, while imperfectly correlated with behavioral social cognition, nonetheless carry predictive validity for observable empathic expression. This finding is included here because it demonstrates, in a controlled non-clinical setting, that self-referential judgments about one’s own empathic capacity can predict actual empathic behavior—a principle that, if operative in clinical populations, would have direct implications for understanding how SRP dysfunction compromises social functioning in psychiatric disorders.

Given that empathy impairments are associated with poor social functioning in schizophrenia and are increasingly recognized as clinically relevant features of psychiatric illness, interest in empathy-focused interventions is growing (42). Such interventions aim not only to improve patients’ understanding of others’ emotional states but also to foster context-appropriate emotional responses. Enhancing empathic capacity may therefore represent a critical therapeutic target for improving functional outcomes across diverse mental disorders.

### 4.3 The modulatory role of empathy in self-related biases

Empathy is integral to the modulation of self-related biases and shapes individuals’ self-perception in relation to others. Desebrock et al. (43) provide important insights into this phenomenon, demonstrating that empathy is a significant predictor of self-bias: individuals with higher trait empathy show more pronounced self-biases in manual motor responses. This finding challenges the prevailing view that self-biases operate independently of higher-level self-related constructs and instead suggests that explicit representations of self–other relations—a core component of self-referential processing—can directly modulate these biases (43).

Supporting this perspective, Wang et al. (44) examined the impact of altering self-construals on in-group biases in empathic responding. The findings indicate that priming an independent self-construal can reduce racial in-group biases in neural responses to others’ suffering. These results suggest that empathy, as modulated by self-construal, can significantly influence the degree of self-related bias, particularly in the context of racial in-group and out-group interactions (44).

Furthermore, Önal et al. (45) found that empathy and the ability to experience one’s own emotions jointly modified the expression of blatant and subtle prejudice (45). This research indicates that empathy can attenuate the expression of both overt and covert prejudice, particularly when individuals are able to recognize their own emotions. These findings imply that empathy, in conjunction with emotional self-awareness, can reduce self-related biases that manifest as prejudice, thereby fostering more equitable social interactions. Collectively, these studies reinforce the view that empathy functions as a domain-general modulator of self-related biases, spanning sensorimotor, affective, and intergroup levels of processing. The bidirectional SRP-empathy framework advanced in this section is theoretically compelling, but the empirical base supporting it has notable gaps. First, much of the evidence for SRP-empathy co-occurrence derives from cross-sectional correlation studies or case-control comparisons, neither of which establishes mechanistic interdependence. The claim that SRP disruption causally impairs empathic functioning—central to simulation theory—requires experimental paradigms in which SRP is directly manipulated (e.g., via neuromodulation or priming) and empathic outcomes are subsequently measured. Such studies remain rare. Second, the Bonfils et al. (16) moderation finding in Section 4.2, while theoretically informative, is based on a small sample (N = 54) and has not been independently replicated; this finding requires replication before it can be used to anchor clinical recommendations. Third, the use of ASD findings to support simulation theory arguments (Section 4.1) involves cross-population generalization that is not empirically validated for schizophrenia, depression, or anxiety; this inference should be treated as a theoretical proposal rather than established evidence. Fourth, the constructs of self-reported empathy and behaviorally assessed social cognition show only weak convergent validity (17), raising questions about which operationalization of empathy is most relevant to SRP dysfunction in clinical populations.

## 5 Empathy-based interventions in psychiatric disorders

### 5.1 Schizophrenia: MERIT, oxytocin, art therapy

**Figure 2:**
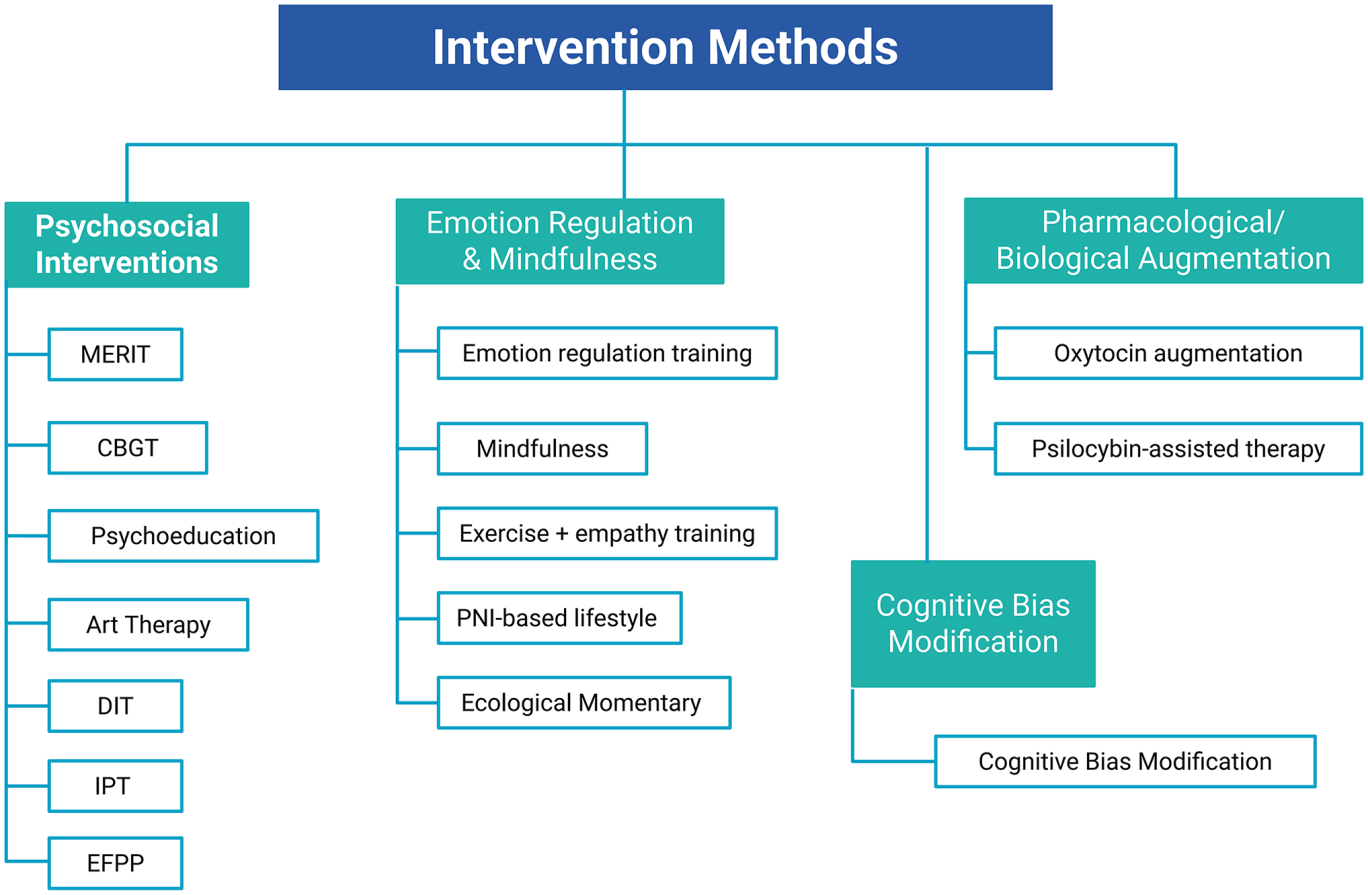
Integrative schematic synthesis of proposed relationships among empathy deficits, self-referential dysfunction, and candidate intervention targets in schizophrenia. This figure is intended as a heuristic framework that synthesizes the theoretical and empirical threads reviewed in Sections 4 and 5, rather than a mechanistic model directly established by empirical testing. Solid arrows represent relationships for which direct empirical evidence exists (cited in the corresponding text sections); dashed arrows represent theoretically proposed but empirically untested links. The reader is referred to the main text for a detailed discussion of the strength of evidence supporting each pathway.

Schizophrenia is associated with marked empathy deficits that substantially impair social functioning, making targeted interventions a clinical priority (42, 46). These impairments may be further compounded by reduced sensitivity to highly expressive emotional cues and by underlying neurobiological abnormalities, such as impaired white-matter integrity (42, 47). **Figure 2** provides an integrative schematic synthesis of the proposed relationships among these empathy deficits, self-referential dysfunction, and the candidate intervention targets discussed in this section. It should be noted that this figure represents a conceptual framework derived from the literature reviewed herein, and not all depicted pathways have been directly tested in a single empirical study.

Several therapeutic strategies show potential in addressing these deficits. MERIT is a psychosocial intervention specifically designed to enhance metacognitive capacity in individuals with schizophrenia. It should be noted that MERIT’s primary therapeutic target is metacognitive capacity—particularly the ability to form integrated representations of one’s own and others’ mental states—rather than SRP as operationally defined in Section 1.1. However, metacognitive improvement plausibly engages self-referential mechanisms, insofar as reflecting on one’s own mental states constitutes a form of deliberate self-referential activity. The relevance of MERIT to the present review therefore rests on this theoretical linkage rather than on direct evidence that MERIT normalizes SRP as indexed by standard paradigms such as the SRET. MERIT posits that improving the ability to reflect on mental states fosters self-directed recovery and enhances social functioning. MERIT is delivered in an interpersonal context that provides a safe and supportive environment for patients to explore their thoughts, feelings, and beliefs about themselves and others. Through guided discovery, therapists help patients develop a more nuanced and integrated understanding of their own mental experiences. This process can reduce symptoms and improve quality of life. The therapeutic effects of MERIT may partly reflect improvements in metacognitive abilities that are closely related to self-referential and social-cognitive functioning. Although the underlying neural mechanisms remain to be clarified, MERIT is conceptually relevant to systems supporting self-processing and understanding of others. This, in turn, may improve both self-awareness and the ability to understand others’ perspectives. While more research is needed to fully elucidate the neural mechanisms of MERIT, the existing evidence suggests that it is a candidate intervention warranting further investigation for addressing the core social cognitive deficits of schizophrenia. Future research should focus on optimizing the delivery of MERIT and identifying the patient characteristics that predict a positive response to this therapy (14).

Oxytocin, a neuropeptide that plays a key role in social bonding and affiliation, has emerged as a potential pharmacological approach to enhancing empathy in schizophrenia. Some studies suggest that intranasal oxytocin may improve selected aspects of social cognition in schizophrenia, including emotion recognition and related social-cognitive functions. These effects have been hypothesized to involve oxytocin’s influence on neural circuits implicated in social processing, possibly including modulation of amygdala and mPFC activity, although the specific neural pathways have not been consistently demonstrated across studies. If such modulation occurs, oxytocin may help create a more positive and receptive social mindset, thereby facilitating empathic engagement. However, although some findings from oxytocin studies are encouraging, there are also inconsistencies and challenges that need to be addressed. The effects of oxytocin may depend strongly on individual factors, such as baseline social-cognitive abilities and genetic variation in the oxytocin receptor gene. Furthermore, the long-term effects of repeated oxytocin administration remain poorly understood. Therefore, oxytocin is currently being investigated as an adjunctive treatment to be used in combination with psychosocial interventions such as MERIT or cognitive-behavioral therapy (CBT). One rationale for adjunctive use is that oxytocin might enhance patients’ receptivity to the therapeutic effects of psychosocial interventions, although whether this translates into more durable improvements in social functioning remains to be established in controlled longitudinal studies. (48, 49).

Furthermore, art therapy provides a non-verbal and creative avenue for individuals with schizophrenia to explore and express their emotions, which can be particularly beneficial for those who struggle with verbal communication and emotional awareness. The process of creating art can help patients externalize their internal experiences, making these experiences more tangible and easier to understand. This can foster greater self-awareness and a more integrated sense of self. Art therapy can also facilitate social interaction and communication, as patients share their artwork with others and discuss the thoughts and feelings it represents. It has been proposed that the therapeutic benefits of art therapy may partly operate through processes relevant to ER and self-referential processing, although direct neuroimaging evidence for this claim is currently lacking. The creative process has been compared to aspects of mindfulness practice, in that it may encourage patients to focus on the present moment and potentially reduce engagement with ruminative thought patterns. It may also provide opportunities for emotional expression, allowing patients to externalize and explore difficult emotions in a supportive environment. Although more research is needed to fully elucidate the mechanisms of art therapy, existing evidence suggests that it may serve as a useful adjunctive approach for schizophrenia, particularly for improving emotional expression, self-awareness, and social functioning, although controlled trials with larger samples are needed to confirm these observations. (50, 51).

Beyond these approaches, improving ER through Ecological Momentary Intervention and psychoeducational programs have demonstrated benefits for social functioning and treatment adherence (52–54). Importantly, the therapist’s empathic attunement has been identified as one component of the therapeutic alliance and a factor that influences treatment engagement and outcomes (55). This finding is relevant to patient SRP in a specific sense: a strong therapeutic alliance, partly constituted by therapist-expressed empathy, creates the relational conditions under which patients with schizophrenia are more likely to engage in the effortful metacognitive and self-referential tasks central to MERIT and related interventions. In this way, therapist empathy functions as a facilitating context for patient-level SRP rehabilitation, rather than as a therapeutic mechanism in its own right.

In summary, the interventions reviewed above—MERIT, oxytocin augmentation, and art therapy—each target distinct but complementary dimensions of SRP and empathy dysfunction in schizophrenia (**Table 1**). Given the multidimensional nature of these deficits, integration of such approaches represents a clinically rational strategy; however, the empirical evidence for combined protocols remains limited. Controlled trials specifically designed to evaluate multi-component interventions are needed before integrated approaches can be recommended with confidence. Future research should prioritize elucidating the neural mechanisms of each intervention and establishing whether their effects on SRP and empathic functioning are additive or synergistic.

**Table 1:** Summary of intervention evidence across disorders and intervention types: methods, targets, and outcomes.

Disorder	Intervention (method/program)	Target (mechanism)	Primary outcomes (with references)
Schizophrenia	MERIT (Metacognitive Reflection and Insight Therapy)	Metacognitive capacity; self/other understanding	Preliminary evidence of improved metacognitive capacity; may support self-directed recovery and social functioning (14).
Schizophrenia	Oxytocin augmentation	Social-emotion circuitry; selected aspects of empathic/social-cognitive processing	Improved selected emotion-understanding or social-cognitive outcomes, although effects are mixed and patient-dependent (48, 49).
Schizophrenia	Ecological Momentary Intervention (EMI)	Emotion regulation; day-to-day symptom management	Improved symptoms and social functioning in schizophrenia-spectrum disorders (53).
Schizophrenia	Psychoeducation	Awareness of deficits; treatment adherence	Improved awareness and treatment adherence; evidence summarized in a systematic review (52).
Schizophrenia	Art therapy	Emotional expression; self-awareness	May improve emotional expression, self-awareness, and social functioning; current evidence is supportive but still limited (50, 51).
Depression	Emotion-regulation training	Reappraisal, adaptive ER skills	Associated with reduced depressive symptoms across therapeutic settings; ER is a plausible empathy-relevant target (56).
Depression	Mindfulness/self-compassion mobile intervention (Serene)	Mindfulness; self-compassion; adaptive ER	Reduced depression, anxiety, and stress; improved adaptive emotion regulation and self-understanding (15).
Depression	Psilocybin-assisted psychotherapy	Rigid self-focus; affective empathy for positive cues	Enhanced affective empathy in patients with major depression; mechanistically relevant to maladaptive self-focus (13).
Depression	Dynamic Interpersonal Therapy (DIT)	Mentalizing capacity; interpersonal meaning-making	Improved mentalizing and depressive symptoms; mediation claim should be interpreted cautiously and tied specifically to DIT evidence (59).
Depression	PNI-based lifestyle intervention	Psychoneuroimmunology-related mind-body regulation	Reduced depression severity and improved spiritual well-being; empathy relevance is indirect (61).
Anxiety disorders	Internet-based Mindfulness Therapy	Mindfulness; anxiety sensitivity	Associated with reductions in anxiety, depression, and insomnia; improved quality of life, with anxiety sensitivity as one candidate mediator (63, 64).
Anxiety disorders	Emotion-Focused Psychodynamic Psychotherapy (EFPP)	Suppressed emotions; interpersonal conflict; emotional awareness and regulation	Reduced anxiety symptoms and improved conflict processing; preliminary evidence from a limited number of studies (66).
Anxiety disorders	Cognitive Behavioral Group Therapy (CBGT) for SAD	Exposure; cognitive restructuring; positive affective empathy	Reduced social anxiety symptoms; empathy-related change has been reported as one mediator in SAD (67).

### 5.2 Depression: targeting emotion regulation and interpersonal processes

Deficits in affective empathy are a recognized feature of depression (13). These deficits contribute to impaired social functioning and reinforce feelings of isolation and distress. Consequently, interventions targeting empathy and its underlying mechanisms have become a focus of therapeutic development. Although **Figure 2** was developed with a schizophrenia focus, the general structure of the proposed SRP-empathy-intervention relationships depicted therein is broadly applicable to the depression interventions reviewed below, with disorder-specific modifications in the nature of SRP dysfunction (negative self-schematic processing rather than self-boundary disturbance) and corresponding intervention targets.

ER skills are a central component of many therapeutic approaches for major depressive disorder. Numerous studies have confirmed that training in ER can significantly reduce symptom severity across various psychotherapeutic frameworks. This is because maladaptive ER strategies, such as rumination and suppression, are known to contribute to both the onset and maintenance of depression. Interventions that teach individuals more adaptive strategies (such as cognitive reappraisal and mindfulness) can help break the cycle of negative affect and cognitive distortions that perpetuate the disorder (56, 57). ER training is included here as an empathy-relevant intervention because the capacity to regulate one’s own affective states constitutes a prerequisite for empathic attunement: individuals who are overwhelmed by their own emotions are less able to respond accurately and flexibly to the emotional states of others. In this sense, ER enhancement does not directly train empathy, but facilitates the emotional availability that underlies empathic engagement.

Moreover, the importance of ER is further underscored by its role as a mediator in other therapeutic interventions. ER skills can strengthen the relationship between mindfulness practice and psychological resilience. This finding suggests that the benefits of mindfulness in depression may be partly mediated by improvements in ER. This indicates that focusing on ER can be a powerful way to strengthen an individual’s adaptive coping capacity. Interventions targeting ER can be delivered in various formats, including individual therapy, group therapy, and digital platforms. These interventions equip individuals with tools to better manage their emotional responses to stressors and negative thoughts. Consequently, they not only alleviate depressive symptoms but also improve overall well-being and reduce the risk of relapse. The emphasis on ER represents a shift towards a more proactive, skill-based approach to treating depression. This approach empowers individuals to take a more active role in their own recovery (58).

Novel biological and digital interventions show promise in modulating empathic and self-referential processes in depression. Psilocybin-assisted psychotherapy has been shown to enhance affective empathy, particularly in response to positive social stimuli, an effect thought to be mediated by disruption of rigid, DMN-anchored self-referential patterns—temporarily attenuating the ruminative self-focus characteristic of depression and facilitating interpersonal reconnection (13). Although this evidence remains preliminary, it positions psychedelic-assisted therapy as a mechanistically distinct complement to conventional pharmacological treatments. Similarly, mobile interventions leveraging self-compassion and mindfulness (e.g., the Serene app) can reduce depressive symptoms by promoting emotional awareness and self-understanding (15).

Psychosocial approaches also target these domains. Dynamic Interpersonal Therapy (DIT) is a time-limited psychodynamic therapy shown to be effective in treating major depressive disorder. A key mechanism of DIT is its focus on enhancing mentalizing capacity—the ability to understand one’s own as well as others’ mental states, including thoughts, feelings, and intentions. Mentalizing is a broader construct than SRP (see Section 1.1): it encompasses both self-directed and other-directed mental state attribution, whereas SRP refers specifically to the process of relating stimuli to the self. Nonetheless, the self-directed component of mentalizing—understanding one’s own thoughts, feelings, and motivations—substantially overlaps with self-referential operations. This focus on mentalizing is particularly relevant to the interplay between self-referential processing and empathy, since both are essential components of mentalizing. DIT helps patients develop a more accurate and nuanced understanding of their own internal world and the internal worlds of others. This improved understanding can alleviate the interpersonal difficulties that often contribute to and are exacerbated by depression. Research has demonstrated that improvements in mentalizing are associated with reduced depressive symptoms in patients receiving DIT, suggesting that mentalizing capacity may represent one therapeutic pathway of this approach (59, 60). However, these findings are correlational in nature; formal mediation would require structural equation modeling or path-analytic testing, which has not yet been conducted in this context. Whether mentalizing improvements statistically mediate DIT’s therapeutic effects therefore remains an empirical question, and this should be treated as a hypothesis rather than an established mechanism. Future research employing mediation analysis designs is needed to substantiate this claim. The therapy provides a safe, structured environment in which patients can explore their interpersonal relationships and practice mentalizing skills in real time. The therapist helps the patient identify their assumptions about others’ intentions and feelings, challenge these assumptions, and consider alternative perspectives. This process can help correct cognitive biases and distortions common in depression, such as the tendency to interpret others’ actions in a negative or critical light. By fostering greater capacity for empathy and self-reflection, DIT can help patients build more satisfying and supportive relationships. In turn, these relationships serve as a buffer against stresses and challenges that might trigger or worsen depressive episodes (59, 60).

Furthermore, psychoneuroimmunology (PNI)-based lifestyle interventions have shown initial evidence of benefit in reducing depression severity and enhancing spiritual well-being. These interventions incorporate techniques such as progressive muscle relaxation and guided imagery, supporting a mind–body integrated approach to treatment (61). It should be noted that PNI-based approaches are not empathy-based interventions per se; and their inclusion requires explicit justification. The rationale is as follows: neuroendocrine and autonomic regulatory processes targeted by PNI interventions—such as cortisol reactivity and vagal tone—are known to modulate the physiological substrates that support social-emotional responsiveness. Because chronic dysregulation of these substrates in depression may contribute to both self-referential biases (e.g., heightened stress-related negative self-focus) and reduced capacity for empathic engagement, PNI-based approaches are relevant to the SRP-empathy framework as interventions that address shared downstream biological pathways rather than SRP or empathy directly. Their primary contribution to this section lies in illustrating the breadth of mind–body mechanisms through which depression-related SRP dysfunction may be addressed.

Combining interventions that target both affective-cognitive and physiological pathways may offer complementary benefits, although direct evidence for such integrated protocols in depression remains limited (62; **Table 1**). This underscores the value of multi-faceted rehabilitation strategies that target both cognitive-affective and physiological pathways.

### 5.3 Anxiety: mindfulness, interpersonal, psychodynamic, and group cognitive-behavioral approaches

Dysregulated self-referential processing and empathic capacity are increasingly recognized as key factors contributing to anxiety disorders. Therapeutic interventions targeting these domains offer multiple pathways to ameliorate symptoms and improve functional outcomes **Figure 2**. 

Internet-Based Mindfulness Therapy has emerged as a modality with preliminary evidence of efficacy to reduce anxiety. A randomized controlled trial involving 91 patients with anxiety disorders showed that a mindfulness intervention led to significant reductions in anxiety, depression, and insomnia symptoms. The intervention also improved patients’ quality of life (63). These benefits may be mediated by a reduction in anxiety sensitivity, which mindfulness training effectively modulates (64).

Interpersonal Psychotherapy offers another treatment avenue for anxiety disorders, although its efficacy varies across different interpersonal problem subtypes. Research on Generalized Anxiety Disorder (GAD) has identified four distinct clusters of interpersonal issues. Although short-term therapy yields overall improvement, effect sizes differ substantially among subtypes. These differences underscore the need for personalized treatment strategies even within diagnostically homogeneous groups (65).

Emotion-Focused Psychodynamic Psychotherapy (EFPP) is a therapeutic approach that integrates techniques from emotion-focused and psychodynamic therapies to address underlying emotional conflicts contributing to anxiety disorders. EFPP is based on the premise that anxiety often stems from suppressed or denied emotions. According to this premise, processing and transforming these emotions may contribute to symptom reduction, although the durability of such relief has not been extensively studied. The therapy provides a safe and supportive environment in which patients can explore their emotional experiences and develop a greater capacity for emotional awareness and regulation. The therapist helps the patient identify and express underlying feelings, and understand how these feelings are connected to their anxiety. Research has shown that EFPP may be effective in resolving intrapsychic and interpersonal conflicts associated with anxiety disorders, though the evidence base remains limited. By helping patients access and process their emotions more adaptively, EFPP has been associated with reductions in symptom severity and improvements in overall functioning in the available studies, though these findings await replication in larger, controlled trials. The therapy’s focus on the emotional roots of anxiety distinguishes it from cognitively oriented approaches such as CBT. These approaches tend to focus on changing maladaptive thought patterns. Both approaches can be effective. However, EFPP may be particularly beneficial for individuals who struggle to identify and express their emotions or whose anxiety stems from early life experiences and relational trauma. By addressing the underlying emotional conflicts that fuel anxiety, EFPP may support the development of a more integrated sense of self, although this proposed outcome has not been systematically assessed using standardized self-referential processing measures (66). It should be acknowledged that the evidence base for EFPP in anxiety disorders currently rests on a limited number of studies, and the mechanistic account presented above—while theoretically coherent—is largely derived from the conceptual framework of the therapy rather than from direct empirical tests of the proposed pathways.

Furthermore, Cognitive Behavioral Group Therapy (CBGT) has particularly benefit for SAD. The group format provides a unique opportunity for individuals with SAD to practice their social skills in a safe and supportive environment. It also allows them to receive feedback from the therapist and their peers. CBGT is based on the principles of CBT. CBT holds that anxiety is maintained by a combination of maladaptive thought patterns (cognitive biases) and avoidance behaviors. The therapy aims to help individuals identify and challenge negative thoughts about themselves and social situations. It also encourages them to gradually confront their fears through behavioral exposure. Recent research suggests that CBGT can alleviate SAD symptoms not only through cognitive restructuring but also by enhancing positive affective empathy. This suggests that the group therapy format may be especially beneficial for individuals with SAD. It provides a context in which patients may become more willing and able to share in others’ positive emotions. By fostering greater positive affective empathy, CBGT can help individuals with SAD develop more adaptive and rewarding social relationships. In turn, these improved relationships can reduce their anxiety and improve their quality of life. The combination of cognitive restructuring, behavioral exposure, and enhancement of empathic skills makes CBGT a comprehensive and powerful intervention for social anxiety disorder (67).

In summary, interventions relevant to empathy, self-processing, and broader social-cognitive functioning may contribute to anxiety reduction through partially distinct mechanisms. Future studies should aim to clarify the neural correlates of these changes and identify biomarkers that predict treatment response (**Table 1**).

The interventions reviewed in Section 5, although variably linked to empathy and SRP, show preliminary promise, but the strength of evidence varies considerably across disorders and treatment modalities. First, many of the studies cited involve small samples, lack active control conditions, or rely on single-site designs, limiting conclusions about efficacy. The oxytocin literature in schizophrenia, while growing, is characterized by inconsistent findings across trials, likely reflecting individual differences in baseline oxytocin receptor sensitivity and social-cognitive profiles—factors that are rarely measured or controlled. Second, the majority of interventions reviewed have not been designed with SRP as an explicit therapeutic target; their inclusion in this review is predicated on theoretical arguments about SRP-empathy mechanisms rather than on trials that directly measure SRP outcomes. Whether these interventions actually normalize SRP—as indexed by SRET performance or DMN connectivity—remains largely untested and should be a priority for future mechanistic trials. Third, the sustainability of treatment gains is generally poorly documented: follow-up periods in most cited studies are short (typically ≤ 3 months), and it is unclear whether improvements in empathic functioning and SRP are maintained beyond the active treatment phase. Fourth, combining interventions—as proposed in the schizophrenia section—is clinically rational but empirically unvalidated; the assumption that effects are additive or synergistic has not been tested in controlled trials. These limitations collectively indicate that the field is still in an early stage of translating mechanistic SRP-empathy models into evidence-based clinical practice.

## 6 Future directions and clinical implications

Current diagnostic approaches to SRP remain constrained by two major sources of heterogeneity. At the neural mechanism level, although numerous studies implicate abnormal activation in regions such as the mPFC and rACC as markers of aberrant self-referential processing in depression (10, 24), inconsistencies due to variations in sample characteristics and task paradigms hinder reproducibility. This suggests that activity in single brain regions is insufficient as a transdiagnostic biomarker. Conversely, dynamic connectivity patterns within the DMN may offer a more robust neural representation across tasks and samples. Jin et al. (68) reported that patients with post-traumatic stress disorder exhibit elevated static connectivity within the DMN, but identified a reduction in temporal variability as a more sensitive pathophysiological marker. It should be noted that PTSD is etiologically and phenomenologically distinct from depression, and these findings cannot be assumed to generalize without direct replication in depressive populations. Nevertheless, this evidence illustrates the broader principle that dynamic connectivity metrics—rather than static activation in single regions—may provide more sensitive indices for studying SRP-related dysfunction across disorders. Sharaev et al. (69) used dynamic causal modeling to show that during resting-state acquisition, bidirectional effective connectivity between the mPFC and the PCC of the DMN is consistently present across individuals, with the intraparietal cortex exerting a significant driving influence. Karamzadeh et al. (70) were the first to capture in real time, using EEG, the sequential activation dynamics of the DMN during task performance. This provides a technical avenue for cross-context monitoring.

Second, at the behavioral and physiological indicator level, there are no consensus-based, validated quantitative metrics for quantifying self-referential weighting using ERP components or memory/judgment-based behavioral metrics (71). This lack of standardization impedes cross-study comparisons. Future research should leverage computational psychiatry approaches—such as Bayesian hierarchical modeling—to transform multidimensional indicators into comparable “self-referential intensity” parameters. Such standardization is essential for establishing quantifiable diagnostic criteria.

For intervention research, it is imperative to develop more targeted and personalized approaches. Although empathy training demonstrates a moderate overall effect size in meta-analyses, high heterogeneity (I² = 63%) and low evidence quality (72) indicate that its efficacy is moderated by individual metacognitive levels and socio-environmental factors. Thus, precision interventions must concurrently address both individual dynamics and contextual embeddedness. A recent systematic review and meta-analysis reported that the efficacy of empathy training varies across affective, cognitive, motivational, and behavioral dimensions, with socio-oriented approaches showing the broadest and most consistent improvements (73). Nevertheless, the sustainability of these training gains is generally limited, especially for affective and cognitive empathy (72).

Socio-environmental factors also critically shape training efficacy. Although the following examples are drawn from non-clinical populations, they are included here to illustrate how contextual and environmental variables—often overlooked in clinical intervention research—may modulate the very processes that SRP-empathy interventions seek to change. Empirical evidence indicates that social status and power asymmetries modulate empathic neural responses. Specifically, individuals in lower hierarchical positions show heightened neural signatures of empathy (74). Moreover, real-time social feedback mechanisms dynamically influence the adaptability of empathic responses (75). In educational settings, contextually embedded and interactive pedagogical strategies significantly enhance students’ empathic competence and communication self-efficacy (76). These findings suggest that the efficacy of SRP-empathy interventions in psychiatric populations may be moderated by patients’ social environments and interpersonal contexts—a consideration that warrants systematic investigation in future clinical trials.

For schizophrenia patients with metacognitive deficits and empathy impairments, the MERIT program should be further optimized. Incorporating virtual reality technologies to simulate social scenarios may enhance patients’ self-awareness and empathic abilities. Strengthening long-term tracking and evaluation is necessary to determine the sustained effectiveness and durability of interventions. Future research should investigate how socio-environmental factors (e.g., social support systems) impact self-cognition and empathy. Future research should explore how improving these factors can augment interventions and enhance rehabilitation outcomes.

In conclusion, personalized and integrated intervention strategies tailored to specific psychiatric disorders are essential, based on the specific characteristics of their self-referential cognitive biases and empathic capacities. These strategies’ cross-diagnostic validity and mechanism specificity must be rigorously evaluated through multicenter randomized controlled trials. This may help move interventions targeting SRP and empathy from empirically informed practice toward more precise and evidence-based clinical approaches.
